# The role of the complement system in traumatic brain injury: a review

**DOI:** 10.1186/s12974-018-1066-z

**Published:** 2018-01-22

**Authors:** Adnan Hammad, Laura Westacott, Malik Zaben

**Affiliations:** 1 School of Clinical Medicine, University of Cambridge, Cambridge, UK; 2 Neuroscience and Mental Health Research Institute (NMHRI), School of Medicine, Cardiff University, Room 4FT 80E, 4th Floor, Heath Park, Cardiff, CF14 4XN UK

## Abstract

Traumatic brain injury (TBI) is an important cause of disability and mortality in the western world. While the initial injury sustained results in damage, it is the subsequent secondary cascade that is thought to be the significant determinant of subsequent outcomes. The changes associated with the secondary injury do not become irreversible until some time after the start of the cascade. This may present a window of opportunity for therapeutic interventions aiming to improve outcomes subsequent to TBI. A prominent contributor to the secondary injury is a multifaceted inflammatory reaction. The complement system plays a notable role in this inflammatory reaction; however, it has often been overlooked in the context of TBI secondary injury. The complement system has homeostatic functions in the uninjured central nervous system (CNS), playing a part in neurodevelopment as well as having protective functions in the fully developed CNS, including protection from infection and inflammation. In the context of CNS injury, it can have a number of deleterious effects, evidence for which primarily comes not only from animal models but also, to a lesser extent, from human post-mortem studies. In stark contrast to this, complement may also promote neurogenesis and plasticity subsequent to CNS injury. This review aims to explore the role of the complement system in TBI secondary injury, by examining evidence from both clinical and animal studies. We examine whether specific complement activation pathways play more prominent roles in TBI than others. We also explore the potential role of complement in post-TBI neuroprotection and CNS repair/regeneration. Finally, we highlight the therapeutic potential of targeting the complement system in the context of TBI and point out certain areas on which future research is needed.

## Background

Traumatic brain injury (TBI) is a leading cause of morbidity in the developed world, with an estimated 1.5–2 million Americans suffering from TBI each year and 52,000 dying as a consequence of TBI per year [1]. While medical advancements, including improvements in pre-hospital and critical care, have contributed greatly to a reduction in TBI-related mortality [2], it is still a leading cause of disability in the developed world, and a significant number of those who suffer from a TBI-related disability may require lifetime care. TBI is defined as damage to the brain resulting from an external force that causes the brain to move quickly within the skull. The damage that results is associated with an altered mental state. TBI can be classified as impact or non-impact. Impact TBI occurs when the head is involved in a direct impact with an object. Non-impact TBI occurs when there is no direct contact between the head and another object, but the head is still exposed to a force, as may occur in acceleration-deceleration injuries. TBI can also be classified on the basis of severity, according to the Glasgow Coma Scale (GCS), as mild, moderate or severe [3].

The initial mechanical impact associated with TBI results in a primary injury, subsequent to which a delayed secondary injury, normally develops [4]. This secondary injury is thought to be an important determinant of outcomes [5]. Thus, there may be a window of opportunity, between the start of the secondary injury cascade and the time at which these secondary changes become irreversible, during which medical intervention (e.g. pharmacologically) may improve outcomes. A major contributor to the secondary injury is neuroinflammation [6]. Microglia resident within the central nervous system (CNS) have been proposed to exist in two main states (M1 and M2), depending on the balance between pro- and anti-inflammatory mediators in the CNS milieu [7], though this view has been recently challenged (e.g. [8]). Pro-inflammatory mediators are believed to favor the M1 phenotype, with M1 microglia playing a crucial role in the clearance of cell debris as well as foreign antigens. However, M1 microglia can also result in damage to healthy cells, as well as releasing further pro-inflammatory mediators that can perpetuate and exacerbate the inflammatory reaction [9, 10]. In contrast, anti-inflammatory mediators are thought to favor the M2 phenotype, which is associated with improved cellular survival and tissue repair [11, 12]. Moreover, in vitro evidence suggests that M2 microglia are able to promote neurite outgrowth [13, 14]. Microglia express receptors for various complement components, including C1q and C3 cleavage products, and thus the complement system plays a crucial role in microglial activation [15]. In pathological states with blood–brain barrier (BBB) compromise, there is evidence that C1q contributes to a shift towards the M1 phenotype [16]. Mannose-binding lectin (MBL), part of the lectin pathway of complement activation, also appears to contribute to microglial activation, perhaps by promoting fibrin deposition [17]. It is thought that both M1 and M2 phenotype switching occurs in TBI, but it appears that there is a bias towards M1 over M2 in TBI secondary injury [7, 13]. In addition to the direct damage induced by M1 microglia, the aforementioned pro-inflammatory cytokines they release can activate astrocytes [10]. While reactive astrocytes can have some protective effects in the CNS, e.g. through the release of neurotrophic factors [7, 18, 19], the major result of astrocytic activation is the formation of glial scars [10]. Glial scars function as barriers to axonal regeneration and extension [7]. The initial injury also compromises the BBB, which permits the entry of peripheral circulating leukocytes, thereby further enhancing the inflammatory response [20, 21]. A major component of the inflammatory response that is often overlooked, the complement system, is also activated as part of the neuroinflammatory response in TBI [22–24]. The endogenous CNS complement system is activated, and its activation is further enhanced by an influx of complement components from the circulation, aided by the breakdown of the BBB.

In addition to the neuroinflammatory response, the secondary injury in TBI is associated with a number of other changes that contribute to damage. Firstly, hypoperfusion of the penumbral region surrounding the core injury occurs. Thus, there is a mismatch between the metabolic requirements of cells in the penumbra and the cerebral blood flow to this area [25]. This mismatch disrupts the sodium-potassium (Na^+^-K^+^) pumps present in neuronal cell membranes, which results in transient depolarization of the cell membrane. Depolarization induces glutamate release [26], which can lead to excitotoxic neuronal death [27]. Excitotoxicity is associated with a rise in intracellular calcium ([Ca^2+^]_i_), which activates a number of enzymes (e.g. proteases, phospholipases and endonucleases) that can damage the cell [28]. The rise in [Ca^2+^]_i_ also enhances the generation of free radicals, which can bring about further damage, including mitochondrial damage that can further exacerbate the oxidative stress [29].

This review will focus particularly on the role the complement system plays during TBI secondary injury. The complement system is now thought to have both deleterious effects and neuroprotective effects in the CNS, and this review will explore the balance between them in the context of TBI secondary injury. The review will also explore the literature concerning attempts to modulate the complement system in the context of TBI secondary injury to explore whether this has had any impact on post-injury outcomes.

### Overview of the complement system

The complement system is traditionally viewed as part of the innate immune system that, along with the adaptive immune system, is involved in protecting the body from foreign antigens [30]. It encompasses more than 30 proteins (both cell surface-bound and soluble) that are zymogens. There is significant amplification in the cascade, as once these zymogens have been activated, the resulting active enzymes can then go on to repeatedly act on their substrates [31].

**Fig. 1:**
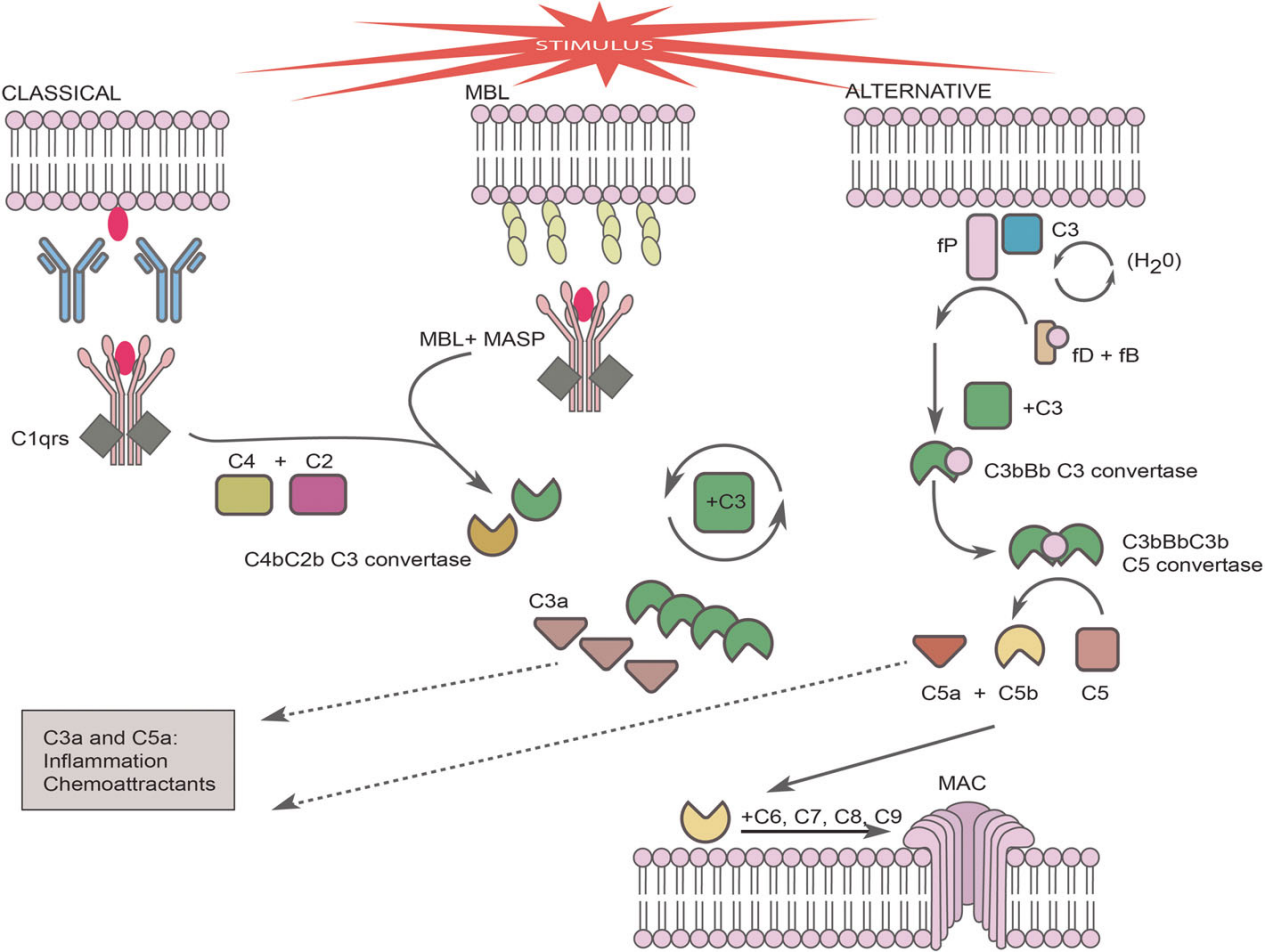
Schematic illustration of the complement cascade. The three activation pathways, the classical, alternative and lectin (MBL) pathways, are all included. Shown are the anaphylatoxins (C3a and C5a), which can trigger mast cell degranulation, and the MAC, which can lead to cell lysis. Adapted from Mathern and Heeger, 2015 [28]

The complement system can be activated via any one of three different pathways: the classical, alternative and lectin pathways [32] (see Fig. 1). Another pathway that can lead to complement activation is the extrinsic pathway of the coagulation cascade [33]. The classical pathway is activated by C1q binding to the fragment crystallizable (Fc) region of IgG and/or IgM antibodies bound to antigens to which they are reactive. C1r and C1s then bind to C1q to form a C1qrs complex, with the C1s component cleaving C4 to C4a and C4b, and C2 to C2a and C2b. C2b can subsequently associate with C4b to form C4bC2b, which functions as a C3 convertase. The C3 convertase cleaves C3 to C3a and C3b, with C3a being an anaphylatoxin. Anaphylatoxins can induce histamine release from mast cells, smooth muscle contraction and can increase vascular permeability. They are also involved in mediating chemotaxis. C3b is an opsonin, which enhances the phagocytosis of apoptotic cells and pathogens. The lectin pathway also generates the same C3 convertase, but via a different mechanism. In this pathway, bacterial carbohydrate motifs are bound by MBL. MBL-associated serine proteases (MASPs) then cleave C4 and C2, generating C4bC2b. The alternative pathway, like the classical pathway, leads to continuous tickover of complement (i.e. its activation at a low background rate). In this pathway, C3 reacts with water to produce C3(H_2_O). Factors B (fB) and D (fD) are then recruited, with fD cleaving fB to generate fBb. fBb can bind to C3b to generate the alternative pathway C3 convertase, C3bBb. The regulator properdin is able to stabilise this convertase [34]. Both convertases, C3bBb and C4b2b, can recruit further C3b molecules and form complexes with them. These complexes, C3bBbC3b and C4b2bC3b, function as C5 convertases. This results in the formation of C5a (which is another anaphylatoxin) and C5b. The formation of C5b can initiate the formation of the membrane attack complex (MAC), by binding to C6, C7, C8 and 10–16 C9 molecules to form C5b-9. The resulting MAC complex can insert into the membranes of cells, resulting in the lysis of non-nucleated cells as well as having milder effects on nucleated cells [31, 35]. C5a is a powerful pro-inflammatory molecule, augmenting the production of various chemokines, cytokines, reactive oxygen species (ROS) as well as mediators such as prostaglandins. It is also capable of acting as a chemoattractant, by upregulating leukocyte adhesion molecules on endothelial cells, thereby enhancing the extravasation of leukocytes into foci of inflammation and/or infection [36]. The complement system also appears to take part in regulating the adaptive immune system, including both T cells and B cells. There is evidence that complement contributes to the regulation of T cell activation and proliferation [37, 38]. Similarly, complement facilitates the activation of B cells and enhances the survival of memory B cells [39–41].

In addition to extracellular complement activation, it has become apparent that the complement cascade can be activated intracellularly too [42, 43]. Human T helper cells store C3 intracellularly. They also contain cathepsin L (CTSL) and C3a receptors (C3aRs). CTSL cleaves C3, generating C3a and C3b. C3a then binds to C3aRs, which has an important function in T cell survival [44]. Since this discovery was made, it has emerged that T cells also possess intracellular C5 stores [43]. Intracellular C5 is cleaved by an as yet unidentified enzyme to generate C5a. C5a can then bind to C5 a receptor 1 (C5aR1), which results in inflammasome activation as well as increased production of ROS by T cells [45].

**Fig. 2:**
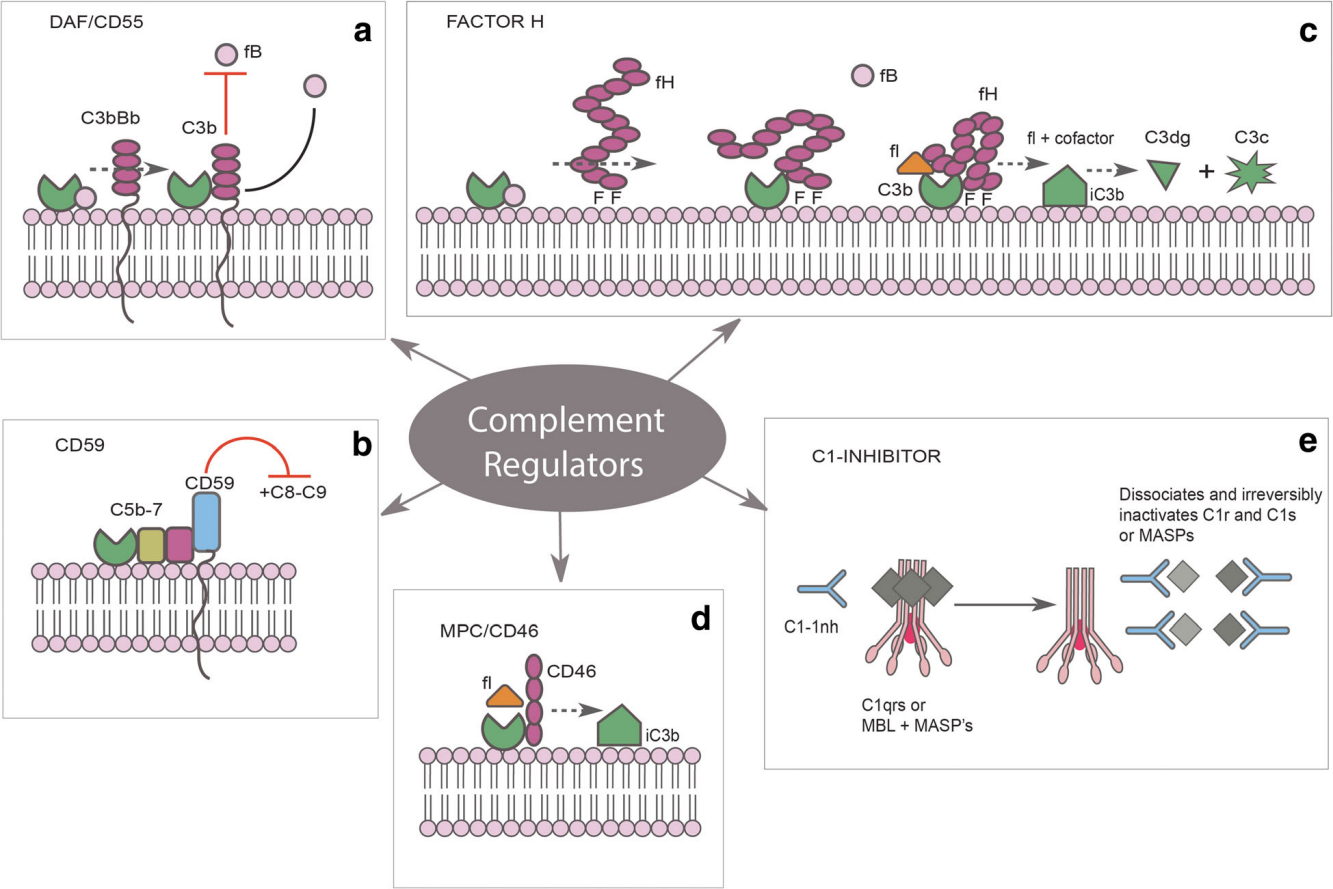
Illustration of the different mechanisms regulating the complement cascade. **a** DAF accelerates the decay of cell surface-assembled classical and alternative pathway C3 and C5 convertases. **b** CD59 inhibits MAC formation. **c** fH accelerates the breakdown of membrane-bound C3b. **d** fI, in concert with MCP, which irreversibly cleaves C3b to iC3b, thereby inactivating it. **e** C1 inhibitor is a protease that inactivates C1r, C1s and mannose-binding lectin-associated serine proteases (MASPs), which irreversibly prevents reformation of the classical and MBL pathways initiating complexes. Adapted from Mathern and Heeger, 2015 [28]

The activation of the complement system must be tightly regulated, in order for it to only be activated when required (i.e. in the presence of a foreign antigen) and so that it is not erroneously activated (see Fig. 2). A number of mechanisms exist that regulate the activity of the convertases. One such mechanism involves decay accelerating factor (DAF), which accelerates the breakdown of both C3 and C5 convertases associated with the cell membrane [46], thereby also interfering with MAC formation [47]. Similarly, serum factor I (fI) in concert with membrane cofactor protein (MCP) acts to cleave C3b to iC3b, which inactivates the C3 convertase [48]. The final mechanism that acts at the C3 convertase stage involves factor H (fH), which binds to C3b attached to the cell membrane and accelerates its breakdown [49]. Further mechanisms that are involved in complement regulation include the inhibition of MAC formation by CD59, and the inhibition of the classical and lectin pathways by C1 inhibitor (C1-INH). CD59 is believed to inhibit MAC formation by blocking the polymerization of C9 and its association with C5b678 [49].

### The complement system in the healthy CNS

Given that the brain and spinal cord are surrounded by the BBB and blood–spinal cord barrier (BSB), respectively, it would be expected that circulating plasma complement components should be mostly excluded from the CNS. However, it has been known for a long time that components of the complement cascade are present within the CNS [50], with a number of studies demonstrating that both neurons and glial cells are capable of synthesizing them [51, 52]. Similarly, a number of studies have shown that complement receptor expression is widespread within the CNS, with mRNA encoding receptors for C3a and C5a being expressed widely [53–55].

The complement system plays a number of vital roles in brain homeostasis [56]. Complement is involved in the clearance of cellular debris as well as cells undergoing apoptosis [56]. It is also thought to play a role in the clearance of amyloid β (Aβ) plaques, deposition of which is associated with ageing and Alzheimer’s disease, via opsonization of the deposited proteins thereby rendering them more susceptible to phagocytosis by resident microglia [57]. Moreover, the complement system has been implicated in protecting the CNS from infection and inflammation. Experiments carried out in a murine model of pneumococcal meningitis have demonstrated that mice lacking either of complement components C1q or C3 are more susceptible to *Streptococcus pneumoniae* infections affecting the CNS compared to controls [58]. Similarly, C3a/glial fibrillary acidic protein (C3a/GFAP) mice, which have restricted overexpression of C3a in the CNS, were less susceptible to mortality subsequent to endotoxin/LPS-induced shock than wildtype and C3a receptor (C3aR) deficient mice [59]. In support of these observations, bacterial infection and/or inflammation were found to lead to the upregulation of complement mRNA expression in the CNS [60, 61].

More recently, further roles for the complement system in the CNS under physiological conditions, including developmental functions, have been revealed [56]. Evidence that complement is involved in synaptic pruning during development came primarily from murine studies on the development of the retinogeniculate pathway. These studies demonstrated that complement components C1q and C3, released by astrocytes, opsonize synapses that are to be eliminated thereby rendering them more susceptible to phagocytosis by microglia [62–64]. C1q^−/−^, C3^−/−^ and C4^−/−^ mice all possessed excess synapses and showed reduced synaptic pruning compared to wildtypes [62, 65, 66]. C1q knockout (C1q^−/−^) mice have a greater incidence of epileptogenesis than wildtypes, as indicated both by epileptiform activity on electroencephalogram (EEG) as well as behavioral seizure activity [64]. Complement also appears to be involved in cerebellar development, for which there is evidence from experiments in neonatal rats. It was found that the expression of both C3aRs and C5a receptors (C5aRs) increased postnatally in cerebellar granule cells, reaching a peak on postnatal day 12 (P12) [67]. Moreover, administration of a C3aR agonist to neonatal rats was associated with an increase in the thickness of the internal granule cell layer and a concomitant decrease in the thickness of the external granule cell layer. This suggests that C3a might normally be involved in facilitating granule cell migration from the external to the internal granule cell layer. In contrast, C5aR agonist administration was associated with an increase in the thickness of the external granule cell layer. It was also associated with enhanced survival of granule cells, secondary to a reduction in the activity of caspase-9, which functions as an apoptosis initiator [67, 68].

### The complement system and CNS injury

When the CNS is subjected to an insult that results in injury, a cascade of secondary pathophysiological events is induced, a key part of which is a prominent neuroinflammatory response. These secondary events compromise the integrity of the neurons present in the penumbral region, which would not have been directly affected by the primary insult to the injured core [69]. Much attention has been directed at the role of the adaptive immune response in secondary injury (e.g. [70]), but there has been more recent interest in the complement system as an important player in secondary injury [71, 72].

#### Clinical studies

Traumatic injuries to both the brain and spinal cord are accompanied by a breakdown of the BBB and BSB, respectively. Therefore, in addition to the fairly low levels of complement components expressed endogenously in the brain/spinal cord, there is a massive influx of serum proteins, including complement components (see Table 1 for a summary). There is also an influx of various innate and adaptive immune cells that are able to contribute to complement activation [69]. The complement system appears to play a particularly significant role in the secondary injury that occurs in the context of TBI. Frontal and temporal lobes resected from TBI patients were probed by immunohistochemistry for the presence of complement components. It was found that the levels of C1q, C3b, C3d and MAC were elevated in the penumbral regions of the injured area compared to controls [23]. Similar results were observed in animal models of TBI [22]. These findings were supported by the observation that a number of complement components, e.g. C3, C1q and fB, were raised in the cerebrospinal fluid (CSF) of TBI patients compared to controls [73, 74]. Similarly, MAC levels were significantly raised in the CSF of TBI patients compared to controls [24]. Moreover, MBL immunostaining in TBI patients was observed around blood vessels in brain tissue that had undergone TBI injury, with no staining observed in controls [75].

#### Animal studies

Evidence for complement involvement in TBI secondary injury comes from a variety of animal models (see Table 2 for a summary). While the majority of the studies discussed below use various models of TBI, two make use of intracerebral hemorrhage (IHC), which is a model of hemorrhagic stroke. Therefore, the degree to which the results of these two experiments are generalizable to TBI is open to debate. The other models used to mimic TBI; cryoinjury, controlled cortical impact (CCI) and standardized weight drop, also vary in their ability to mimic various aspects of TBI. For example, while cryoinjury induces cerebral edema and BBB compromise, both of which are features of TBI [76], the mechanism of injury is not mechanical as in TBI. In contrast, standardized weight-drop models more faithfully mimic the mechanical injury seen in TBI, but they have the disadvantage of low reproducibility/reliability [77]. Therefore, the applicability of these models to human TBI is debatable, and the interpretation of results obtained using such models in the context of human TBI should only be done with great care. CCI, like standardized weight-drop models, mimics the mechanical mechanism of injury of TBI, and has the advantage over other TBI models of versatility, such that the depth and velocity of the impact can be controlled more easily. The results are therefore more highly reproducible [77]. Moreover, the pathological changes seen with this model closely mimic those seen in TBI itself [78]. Therefore, results obtained using this model may be more applicable to humans, but care should still be taken even when drawing conclusions from studies based on this model.

The earliest animal study investigating the involvement of the complement system in TBI secondary injury involved the administration to rats of soluble complement receptor 1 (sCR1), which, by suppressing C3 convertase formation, inhibits the classical, lectin and alternative complement activation pathways. Rats treated with sCR1 had reduced brain neutrophil infiltration compared to vehicle-treated rats, suggesting that complement plays a pivotal role in the neuroinflammatory response induced by TBI. [79] These findings were supported by studies performed on C3 null (C3^−/−^) mice. When these mice were exposed to an ICH injury, reduced leukocyte infiltration, microglial activation and edema build-up were observed in the penumbral region surrounding the site of injury, when compared with control mice. This was paralleled by a reduced motor deficit in the affected limb compared to controls [80]. Similar results were obtained with C3 null mice exposed to cryoinjury [81]. In a CCI model of TBI, C3^−/−^ mice were found to have reduced brain leukocyte infiltration compared to wildtypes, but there were no differences between them with regard to injury size or neurological deficits [82]. CNS-restricted overexpression of complement receptor type 1-related protein y (Crry), which inhibits C3 convertase formation, resulted in better neurological outcomes when compared with control mice, even up to 4 weeks after the initial injury [83]. Based on these results, recombinant Crry (Crry-Ig) was administered to mice that had undergone a form of closed head injury. Reduced tissue loss as well as improved neurological outcomes was found in these mice, compared with vehicle-treated mice that had undergone the same type of injury, when Crry-Ig was administered 1 and 24 h after the initial injury [84].

Animal models have also been used to investigate the role of the anaphylatoxins (i.e. C3a and C5a) in TBI secondary injury. Interfering with the function of C5a, e.g. in C5 null (C5^−/−^) mice or by administering an antagonist of C5aR, reduced secondary damage in a cryoinjury model of TBI [81]. Furthermore, administration of another C5aR antagonist, [hexapeptide-derived macrocycle AcF (OPdChaWR)], in an ICH model was found to improve spatial memory (as measured by performance in the Morris water maze) as well as general neurological function, when compared with vehicle-treated controls. This was paralleled by a reduction in leukocyte infiltration and edema in the vicinity of the lesion [85]. When the C5aR antagonist was combined with a C3aR antagonist, SB290157, a synergistic neuroprotective effect was observed [85]. However, the fact that SB290157 can also function as a C3aR agonist in particular cells, thought to have a high density of C3aRs, complicates the interpretation of the aforementioned result [86, 87].

Similarly, animal studies have revealed a prominent role for the MAC in secondary injury following TBI. The role of the MAC was investigated using CD59 null (CD59^−/−^) mice, which displayed increased MAC attachment to cell membranes due to the absence of the MAC regulatory protein, CD59. When these mice were exposed to a focal closed head injury, they displayed increased neuronal loss and worse neurological outcomes when compared to controls that had undergone the same injury [88]. In contrast, interfering with MAC formation by administrating OmCI, a complement inhibitor that binds C5, was found to reduce neuronal death, microglial activation and neurological deficits in a TBI mouse model when compared with vehicle-treated controls. Administration of a C6 antisense oligonucleotide, which blocks MAC formation by inhibiting C6, yielded very similar results [89]. A recent study building on these findings generated an inhibitor of MAC complex formation, composed of complement receptor of the Ig superfamily (CRIg) fused with CD59 (CD59-2a-CRIg). Administration of this inhibitor to a TBI mouse model was found to reduce MAC formation, neuronal damage and microglial activation compared to vehicle-treated controls. Paralleling this, it was found that neurological outcomes were significantly better in the mice treated with this inhibitor when compared with placebo-treated mice [90].

#### Animal studies focusing on specific complement activation pathways

Attempts to tease out the relative contributions to TBI secondary injury of the different complement activation pathways have been made using animal models [76] (see Table 2 for a summary). Mice lacking fB (fB^−/−^), whose alternative pathway of complement activation is non-functional, were found to have reduced neuronal loss with concomitant upregulation of the anti-apoptotic regulatory protein Bcl-2 and downregulation of the pro-apoptotic Fas receptor, compared with control mice subsequent to TBI [91]. Extending this, a study investigated the effect a monoclonal antibody directed against fB (mab1379) had when administered to control mice, which were exposed to a TBI-like injury, 1 or 24 h after injury. These mice displayed reduced neuronal loss, an attenuated inflammatory response, as well as upregulation of genes associated with neuroprotection when compared with vehicle-treated mice [79]. Interestingly, however, there was no difference observed between the two groups in terms of neurological function [92]. Experimental evidence has also implicated both the classical and lectin pathways in the pathogenesis of TBI. Mice lacking complement component C4 (C4^−/−^), which is involved in both the classical and lectin pathways, and wildtype mice were subjected to a CCI injury. C4^−/−^ mice showed decreased brain tissue damage and reduced motor deficits, compared to wildtypes, after CCI. These improvements were reversed if recombinant human C4 was administered to C4^−/−^ mice. Similarly, it was found that mice given C1-INH (which inhibits both the classical and lectin pathways) 10 min after the initial injury developed smaller contusions and had reduced cognitive and motor dysfunction compared to vehicle-treated controls. Delayed administration of C1-INH (60 min post-injury) led to a reduction in motor dysfunction, but had no effect on cognitive deficits or contusion size [93]. The involvement of the lectin pathway in TBI has been further investigated by immunostaining for MBL-A and MBL-C in the cortex of wildtype mice post-CCI. MBL-C immunostaining was more intense 30 min post-injury, and this lasted for a further week. MBL-A immunostaining was less prominent. Neuronal loss in MBL-A/MBL-C knockout (MBL−/−) mice was reduced when compared with wildtypes 5 weeks post-injury, which was paralleled by a reduction in sensorimotor impairment when assessed 2–4 weeks post-lesion [75]. In contrast to this, early after a CCI injury (6 h post-injury), increased neurodegeneration was observed in the hippocampi of MBL^−/−^ mice when compared with WT mice. Neurological deficits in MBL^−/−^ mice were also greater than those in WT mice, when assessed a week after injury [94]. Thus, it may be that the lectin pathway functions in a neuroprotective capacity in the early phase of TBI secondary injury, before switching to a deleterious phenotype in the late phase.

### The complement system and CNS repair

**Fig. 3:**
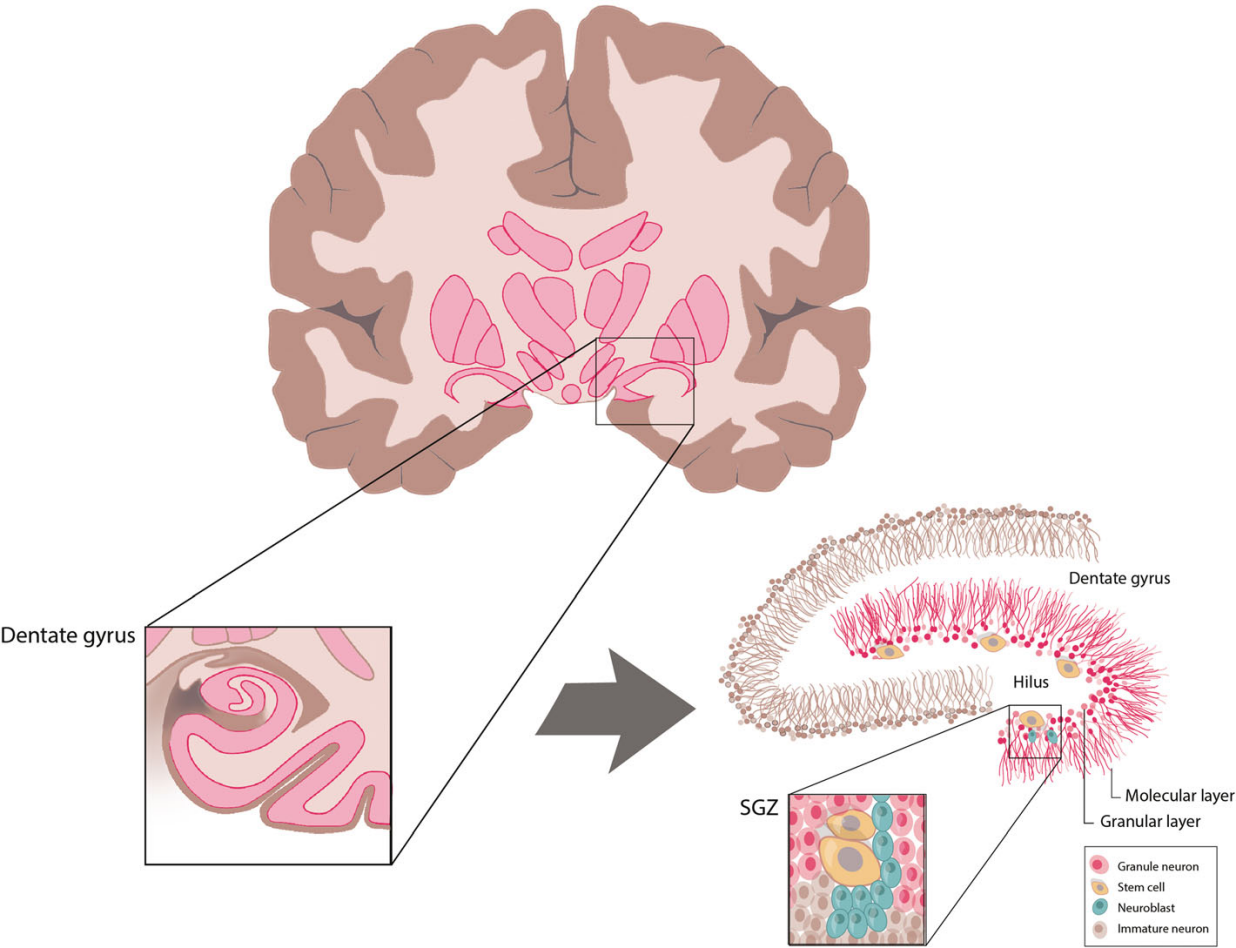
Subgranular zone (SGZ) neurogenesis in the dentate gyrus (DG) of the hippocampus of the adult human brain. Coronal section (top left) through the human brain, illustrating the location of the hippocampus within the medial temporal lobe, and zoomed in view of the hippocampal formation, demonstrating its structure. Illustration of the finer structural details of the hippocampal formation (bottom right), with neural stem cells (NSCs) shown present within the SGZ. Adapted from Vescovi, Galli and Reynolds, 2006 [126]

**Fig. 4:**
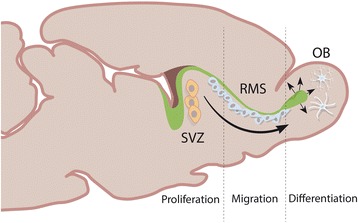
Neurogenesis in the subventricular zone (SVZ). Sagittal section through the rodent brain, illustrating how NSCs generated in the SVZ migrate via the rostral migratory stream (RMS0 to the olfactory bulb (OB)). As the cells migrate towards the OB, they undergo terminal differentiation. Adapted from Abrous, Koehl and Moal, 2005 [127]

While activation of the complement system can have a number of deleterious effects in the CNS, there is evidence that it also plays a prominent role in CNS repair, protection and regeneration [95]. For example, the complement system has been implicated in neurogenesis. In the adult human, neurogenesis is believed to take place in two brain regions: the subgranular zone (SGZ) of the dentate gyrus (DG) of the hippocampus (see Fig. 3) [96] and the subventricular zone (SVZ) [86, 87] (see Fig. 4). The neuroblasts generated in the SVZ migrate, via the rostral migratory stream (RMS), to the olfactory bulb (OB) [97, 98]. Neural precursor cells (NPCs) have been shown to express C3aRs and C5aRs [99]. In vitro studies have demonstrated that the application of C3a to NPCs enhances their maturation and migration [100]. In vivo studies have also shown that administration of a C3aR antagonist to mice results in reduced neurogenesis in the SGZ, the SVZ and the OB. Put together, these findings suggest a role for C3a/C3aR in neurogenesis. Interestingly, despite the fact that SVZ NPCs appear to express C5aRs, C5a signaling through C5aRs was not thought to play a role in SVZ neurogenesis [101], although more recent evidence suggests that C5aR1 signaling drives mouse embryonic neural progenitor cell proliferation in the SVZ by signaling via protein kinase C ζ (PKCζ) [102]. In contrast, complement receptor 2 (CR2), which is expressed by DG progenitor cells, appears to inhibit neurogenesis. Evidence for this comes from CR2-deficient mice, which show increased neurogenesis, whereas C3d and interferon-α (IFN-α) (both of which bind CR2) are associated with downregulation of neurogenesis [103].

Furthermore, evidence for a role of complement in neurogenesis induced by brain injury has emerged from both human and animal studies. In human TBI cases, it was found that neural stem cell (NSC) markers were upregulated in the cortex surrounding the lesion compared to controls, indicating that neurogenesis is induced in response to TBI [104]. A comparison between C3 null mice and control mice that were subjected to a transient ischemic insult revealed a significant reduction in SVZ neurogenesis 7 days post-insult in the C3 null mice. These differences were not linked to reactive gliosis, as there was no difference between the C3 null and control mice in that regard [99]. Further evidence linking C3a to neurogenesis in the context of brain injury came from experiments in ischemic neonatal mice. C3a/GFAP mice subjected to neonatal hypoxic ischemia (HI) demonstrated preserved hippocampal volume and greater levels of adult born neurons in the DG compared to wildtypes. C3a therefore exerted a protective effect on adult neurogenesis in the context of ischemia. Furthermore, as adults, animals treated with C3a peptide shortly after neonatal HI demonstrated improved memory in a cue-induced fear conditioning paradigm. In contrast, administration of C3a peptide to C3aR null ischemic neonatal mice had no effect on the aforementioned parameters [105]. Consistent with the improvement in memory function observed with C3a administration in wildtype mice is the finding that adult neurogenesis in the SGZ leads to improved learning and memory function [106–109].

A number of experiments also indicate that the complement system can have neuroprotective capabilities in the CNS [95]. Rat neurons exposed to C1q survived longer than neurons not treated with C1q, and they had a statistically greater number of neurites [110]. These neuroprotective effects of C1q may be due to upregulation of cytoskeletal genes involved in synaptic function, increased metabolism of cholesterol, as well as upregulation of neurotrophic factors including neurotrophin 3 and nerve growth factor (NGF) [111]. Exposure of C5-deficient mice to kainic acid (KA), which can act as an excitotoxin (with excitotoxicity being one of the likely mechanisms of secondary injury in TBI), resulted in greater neuronal death than in wildtype mice exposed to the same insult [112]. Thus, it appears that C5 may play a role in protecting neurons against death by excitotoxicity. A further study demonstrated that it is the C5a fragment, more specifically, that plays an important neuroprotective role. Administration of C5a with KA was found to result in reduced neuronal death when compared with KA alone. Further analysis suggested that C5a achieves this via a signalling cascade that results in caspase-3 downregulation, thereby reducing apoptotic cell death [113]. C3a appears to have similar neuroprotective effects to C5a [95]. In in vitro experiments where neuronal-astrocytic co-cultures were exposed to N-methyl-D-aspartate (NMDA), which can function as another excitotoxin, concomitant application of human C3a resulted in reduced neuronal death relative to NMDA application alone. This neuroprotective effect, while facilitated by C3a, appeared to be mediated by the astrocytes, as it was abrogated in pure neuronal cultures (i.e. in the absence of astrocytes) [114]. While the MAC complex at higher concentrations promotes cell lysis, it appears to have neuroprotective properties at sublytic concentrations [95]. It can promote the survival of oligodendrocytes within the CNS by inhibiting their apoptosis. It was found that this effect was mediated secondary to inhibition of pro-apoptotic factors (e.g. caspase-3) and converse activation of anti-apoptotic factors (e.g. Bcl-2) [115]. Enhanced oligodendrocyte survival should reduce the extent of demyelination subsequent to an injury to the CNS, thereby aiding in the maintenance of axonal integrity. Pharmacological inhibition of phosphoinositide 3-kinase (PI3K) was found to partially reverse the pro-survival effect of sublytic concentrations of MAC on oligodendrocytes, which suggests that such concentrations of MAC may mediate these effects via a PI3K signaling pathway [116].

Furthermore, CNS glia, including microglia and astrocytes, secrete a number of neurotrophic factors in response to stimulation by complement components [95]. Consistent with this, astrocytes are known to produce NGF subsequent to CNS injury, in response to complement components (C3a and C5a) as well as cytokines (interleukin-1β) [117, 118]. Similarly, C3a has been shown to induce NGF production by microglia [119]. NGF is known to promote the survival of neurons (including cholinergic neurons) subsequent to axotomy, as well as promoting sprouting and regeneration [120]. In addition to its role in promoting neuronal regeneration, complement has been implicated in promoting not only just oligodendrocyte survival, but also proliferation, subsequent to CNS injury [121]. The mechanism appears to involve sublytic doses of the MAC inducing the oligodendrocyte cell cycle [122], by inducing an array of mitogenic and anti-apoptotic signaling pathways [123, 124]. Enhanced oligodendrocyte proliferation would be expected to enhance neuronal myelination, thereby contributing to the maintenance of axonal integrity. Experiments comparing C5-deficient to C5-sufficient experimental autoimmune encephalomyelitis (EAE) mice, a multiple sclerosis (MS) model, have lent support to this notion. C5-deficient mice displayed marked reactive gliosis and Wallerian degeneration of axons. This was in stark contrast with C5-sufficient mice that were found to have significantly reduced Wallerian degeneration linked to concurrent remyelination and reduced gliosis [125]. Therefore, C5 appears to play an important facilitatory role in remyelination subsequent to brain injury, which can contribute to improved neuronal survival.

### Future perspectives

A lot of progress has been made, over the past 30 years or so, in determining the role the complement system plays in TBI secondary injury. However, a multitude of questions that must be addressed by future research remain. The complement system can have both neurotoxic effects as well as neuroprotective influences subsequent to CNS injury. While the complement system appears to play a role in neurogenesis and promoting neuronal survival, including subsequent to CNS injury (e.g. in the context of excitotoxicity and experimental MS), there are few studies that have investigated this in the context of TBI secondary injury. A goal of future research should, therefore, be to address directly whether complement is involved in promoting neurogenesis and neuroprotection subsequent to TBI, e.g. by investigating this in animal models of TBI (e.g. standardized weight-drop and CCI models) as well as human TBI patients. If there is indeed evidence for a neuroprotective role of complement in TBI, then it may be possible to manipulate the complement system (e.g. pharmacologically) to promote neurogenesis/regeneration, which may in turn ameliorate the disabilities that often result from TBI. Complement may play a part in mediating plasticity after TBI, as it is known to have a developmental role in synaptic pruning, but this has not been investigated either in human TBI patients or in animal models of TBI. Thus, a future goal would be to address this, as this could provide another future target for manipulation in order to improve neurological outcomes after TBI. It would also be instructive to carry out studies that are designed to assess a greater number of endpoints, as certain complement components may function neuroprotectively early on during secondary injury but may then switch to become harmful, or vice versa. Therefore, it would be important to ascertain timelines for the actions of the different complement components, in order to work out how best to manipulate them (i.e. whether to activate them or inhibit them) and when best to do so (i.e. whether early or late in the course of the secondary injury). Finally, while attempts have been made using animal models to disentangle the influences of each of the complement activation pathways in TBI, more studies (including ones in human patients) will be required in order to shed more light on the contributions each makes to the pathophysiology of TBI.

### Clinical and translational perspective

Based on current evidence in the literature, which has mainly focused on the deleterious effects of complement in the context of TBI, it appears that complement functions in a mainly damaging fashion in TBI. As discussed in the section entitled ‘The complement system and CNS injury’, dampening down the activity of the complement system, by various means in animal models of TBI, has been generally associated with reduced lesion sizes as well as improved neurological outcomes. Thus, it would appear that a promising future strategy for TBI management would be to target the complement system for downregulation, e.g. by administering pharmacological antagonists of various known mediators in the complement pathway. While pre-clinical studies performed in animal models have yielded promising results (with a number of therapeutics, e.g. sCR1, Crry-Ig, and SB290157, displaying some efficacy), further experiments are required to determine which pharmacological agents would be most appropriate, what doses they must be administered at, via which route(s) they must be administered, and when after the initial injury it would be best to administer them. Subsequent to that, human trials must be started in order to determine whether such agents would be both safe and useful in the target patient group.

## Conclusion

TBI is a leading cause of morbidity in the western world. While healthcare advancements have resulted in a reduction in mortality associated with TBI, it is still a major cause of disability. Neurological outcomes subsequent to TBI are significantly influenced by the secondary sequelae that follow the initial injury. The secondary injury is multifaceted, involving a prominent neuroinflammatory response, in addition to ischemia, excitotoxic neuronal cell death, and free radical production. An important part of the ensuing inflammatory response is activation of the complement cascade. The complement system can have both neurotoxic and neuroprotective effects subsequent to CNS injury. As it takes time for the secondary sequelae of TBI to develop and for any resulting changes to become irreversible, this may provide a window of opportunity for interventions that may improve outcomes subsequent to TBI. One promising target for such interventions is the complement system. Pharmacological agents targeting components of the complement system have been trialed in animal models, with some promising results. Pending further investigations, it may be possible to translate such agents to clinical practice in the future, which may revolutionize the management of TBI.

## Abbreviations

BBB: Blood–brain barrier
C5aR1: C5 a receptor 1
CCI: Controlled cortical impact
CNS: Central nervous system
CR2: Complement receptor 2
CSF: Cerebrospinal fluid
CTSL: Cathepsin L
DG: Dentate gyrus
GCS: Glasgow Coma Scale
GFAP: Fibrillary acidic protein
ICH: Intracerebral haemorrhage
IFN-α: interferon-α
KA: Kainic acid
MAC: Membrane attack complex
MASPs: MBL-associated serine proteases
MBL: Mannose-binding lectin
NMDA: N-methyl-D-aspartate
PI3K: Phosphoinositide 3-kinase
PKCζ: Protein kinase C ζ
RMS: Rostral migratory stream
ROS: Reactive oxygen species
sCR1: Soluble complement receptor 1
SGZ: Subgranular zone
SVZ: Subventricular zone
TBI: Traumatic brain injury
