# Correction to: the role of the complement system in traumatic brain injury: a review

**DOI:** 10.1186/s12974-018-1097-5

**Published:** 2018-02-23

**Authors:** Adnan Hammad, Laura Westacott, Malik Zaben

**Affiliations:** 1 School of Clinical Medicine, University of Cambridge, Cambridge, UK; 2 Neuroscience and Mental Health Research Institute (NMHRI), School of Medicine, Cardiff University, Room 4FT 80E, 4th Floor, Heath Park, Cardiff, CF14 4XN UK

## Correction

After publication of the article [1], it was brought to our attention that Tables 1 and 2 were missing from the final manuscript, These tables can be seen below and have now been added to the revised version of the article.

**Table 1:** Summary of clinical studies investigating the role of complement in TBI

Study	Type of brain insult	Controls	Assay	Complement pathway(s)	Relevant finding(s)
Bellander et al., 2001 [23]	Cerebral contusion (*n*=16)	Epileptic patients undergoing hippocampectomy (*n*=3)	Immunohistochemistry (IHC) for C1q, C3b, C3d and MAC; *in situ* hybridization for C3 mRNA	All	Increased immunoreactivity for C1q, C3b, C3d and the MAC in the immediate vicinity of neurons in the penumbra area of the contusion in the patient group. In situ hybridization revealed C3 mRNA in the penumbra.
Kossmann et al., 1997 [74]	Severe TBI (*n*=13): closed head injury (*n*=12); open head injury (*n*=1)	Patients undergoing diagnostic lumbar puncture (*n*=22)	Radioimmunoassay (RIA) or enzyme linked immunosorbent assay (ELISA) for C3 and fB	All	Elevated levels of C3 and fB in CSF of TBI patients compared to controls.
Stahel et al., 2001 [24]	Severe TBI (closed head injury) (*n*=11)	Patients, without any known head trauma or inflammatory neurological disease, undergoing diagnostic spinal tap (*n*=12)	ELISA for sC5b-9	All	Raised mean sC5b-9 levels in CSF of 10 out of 11 TBI patients compared to CSF obtained from controls.
Longhi et al., 2014 [75]	Cerebral contusion (*n*=6)	Non-TBI patients who received surgery for brain tumors (*n*=2)	IHC for MBL	Lectin	MBL-positive staining was observed in brain tissue samples from cerebral contusion patients, but not in equivalent samples from controls.

**Table 2:** Summary of studies investigating the role of complement in TBI using animal models

Study	Species	Model	Treatment	Complement pathway(s)	Relevant finding(s)
Kaczorowski et al., 1995 [79]	Rat	Standardized weight-drop	• WT + sCR1 • WT + vehicle	All	Rats treated with sCR1 had reduced brain neutrophil infiltration compared to vehicle-treated rats, suggesting that complement plays a role in the neuroinflammatory response induced by TBI.
Yang et al., 2006 [80]	Mouse	Intracerebral hemorrhage (ICH)	• C3^-/-^ • WT	All	C3^-/-^ mice, when compared with WT mice, showed reduced brain oedema, lower hemeoxygenase-1 levels, and reduced microglia activation and neutrophil infiltration after injury. The C3^-/-^ mice also displayed reduced forelimb dysfunction in comparison with WT mice.
Sewell et al., 2004 [81]	Mouse	Cryoinjury	• C3^-/-^ • C5^-/-^ • WT + C5aR antagonist • WT	All	Injury size and neutrophil infiltration were significantly reduced in C3^-/-^ mice compared with WT mice. Neutrophil infiltration was also found to be reduced in C5^-/-^ mice and WT mice treated with a C5aR antagonist compared with untreated WT mice.
Rancan et al., 2003 [83]	Mouse	Standardized weight-drop model (focal closed head injury)	• GFAP-sCrry • WT	All	GFAP-sCrry mice were found to have reduced neurological deficits and BBB compromise compared with WT mice exposed to the same TBI-like injury.
Leinhase et al., 2006a [84]	Mouse	Standardized weight-drop	• WT + Crry-Ig • WT + vehicle	All	Administration of Crry-Ig 1 and 24 hrs after injury was associated with a reduction in tissue loss and improvement in neurological function when compared with vehicle-treated mice.
Garrett et al., 2009 [85]	Mouse	ICH	• WT + C5aR antagonist • WT + C5aR antagonist + C3aR antagonist • WT + vehicle	All	Administration of a C5aR antagonist alone, or both C5aR and C3aR antagonists, to WT mice was associated with a reduced deficit in neurological function and reduced spatial memory dysfunction compared with vehicle-treated WT mice. These effects were also associated with a reduction in leukocyte infiltration and oedema A synergistic effect was observed upon administration of both antagonists.
Stahel et al., 2009 [88]	Mouse	Standardized weight-drop	• CD59a^-/-^ • WT	All	CD95a^-/-^ mice had significantly worse neurological outcomes than WT mice 7 days post-injury. Neuronal cell death in CD59a^-/-^ mice was also greater than that in WT mice, as indicated both by serum NSE levels as well as TUNEL histochemistry.
Fluiter et al., 2014 [89]	Mouse	Standardized weight-drop	• WT + OmCI • WT + C6 antisense • WT + vehicle	All	Interfering with MAC formation, by administering OmCI or C6 antisense to WT mice, was associated with a reduction in neuronal death, microglial activation and neurological deficit, compared with vehicle-treated WT mice.
Ruseva et al., 2015 [90]	Mouse	Standardized weight-drop	• WT + CD59-2a-CRIg • WT + vehicle	All	CD59-2a-CRIg was found to inhibit MAC formation *in vitro*. *In vivo* administration of CD59-2a-CRIg reduced MAC formation, neuronal damage, and microglial activation compared to vehicle treated controls. Neurological outcomes were ameliorated with CD59-2a-CRIg administration.
Leinhase et al., 2006b [91]	Mouse	Standardized weight-drop	• fB-/- • WT	Alternative	fB^-/-^ mice were found to have reduced neuronal loss, upregulation of the anti-apoptotic regulatory protein Bcl-2, and downregulation of the pro-apoptotic Fas receptor, compared with WT mice subsequent to a TBI-like injury.
Leinhase et al., 2007 [92]	Mouse	Standardized weight-drop	• WT + mab1379 • WT	Alternative	Administration of mab1379, a monoclonal antibody directed against fB, 1 or 24 hrs after injury was associated with reduced neuronal loss, an attenuated inflammatory response, as well as upregulation of genes associated with neuroprotection, in comparison with vehicle administration.
You et al., 2007 [82]	Mouse	Controlled cortical impact (CCI)	• C3^-/-^ • C4^-/-^ • C4^-/-^ + hC4 • WT	• All (C3) • Classical and lectin (C4)	C3^-/-^ mice were found to have reduced brain leukocyte infiltration compared to WT, but there were no differences between them in terms of injury size or neurological deficits. C4^-/-^ mice showed decreased brain tissue damage and reduced motor deficits, compared to WT, after CCI. These improvements were reversed if recombinant human C4 (hC4) was administered to C4^-/-^ mice.
Longhi et al., 2009 [93]	Mouse	CCI	• WT + C1-INH • WT	Classical and lectin	Mice given C1-INH 10 mins after injury developed smaller contusions, and had reduced cognitive and motor dysfunction compared to vehicle-treated controls. Delayed administration of C1-INH (60 mins post-injury) led to a reduction in motor dysfunction, but had no effect on cognitive deficits or contusion size.
Longhi et al., 2014 [75]	Mouse	CCI	• MBL^-/-^ • WT	Lectin	MBL-C and MBL-A immunostaining was upregulated 30 mins post-injury, and this lasted for a week. MBL-A immunostaining was less prominent. Neuronal loss in MBL-A/MBL-C knockout (MBL-/-) mice was reduced when compared with wildtypes 5 weeks post-injury, which was paralleled by a reduction in sensorimotor impairment when assessed 2-4 weeks post-lesion.
Yager et al., 2008 [94]	Mouse	CCI	• MBL^-/-^ • WT	Lectin	6 hrs post-injury, increased neurodegeneration was observed in the hippocampi of MBL^-/-^ mice when compared with WT mice. Neurological deficits in MBL^-/-^ mice were also greater than those in WT mice, when assessed a week after injury.
